# Area disadvantage and mental health over the life course: a 69-year prospective birth cohort study

**DOI:** 10.1007/s00127-023-02427-x

**Published:** 2023-02-09

**Authors:** Ioannis Bakolis, Emily T. Murray, Rebecca Hardy, Stephani L. Hatch, Marcus Richards

**Affiliations:** 1 Department of Biostatistics and Health Informatics, Institute of Psychiatry, Psychology and Neuroscience, King’s College London, London, UK; 2 Centre for Implementation Science, Health Service and Population Research Department, Institute of Psychiatry, Psychology and Neuroscience, King’s College London, London, UK; 3 Department of Epidemiology and Public Health, University College London, London, UK; 4 Social Research Institute, University College London, London, UK; 5 School of Sport, Exercise and Health Sciences, Loughborough University, Loughborough, UK; 6 Department of Psychological Medicine, King’s College London, IOPPN and South London and Maudsley NHS Foundation Trust, London, UK; 7 ESRC Centre for Society and Mental Health, King’s College London, London, UK; 8 MRC Unit for Lifelong Health and Ageing at UCL, University College London, London, UK

**Keywords:** Birth cohort studies, Mental health, Cross-classified models, Disadvantage, Life course, Area

## Abstract

**Purpose:**

Existing evidence on the mental health consequences of disadvantaged areas uses cross-sectional or longitudinal studies with short observation periods. The objective of this research was to investigate this association over a 69-year period.

**Methods:**

Data were obtained from the MRC National Survey of Health and Development (NSHD; the British 1946 birth cohort), which consisted of 2125 participants at 69 years. We assessed longitudinal associations between area disadvantage and mental health symptoms at adolescence and adulthood with use of multilevel modelling framework.

**Results:**

After adjustment for father’s social class, for each one percentage increase in area disadvantage at age 4, there was a 0.02 (95% CI 0.001, 0.04) mean increase in the total score of the neuroticism scale at age 13–15. After adjustment for father’s social class, adult socio-economic position, cognitive ability and educational attainment, a one percentage increase in change score of area disadvantage between age 4 and 26 was associated with a mean increase in the total Psychiatric Symptom Frequency score (MD 0.06; 95% CI 0.007, 0.11). Similar associations were observed with change scores between ages 4, 53, 60 and total General Health Questionnaire-28 score at age 53 (MD 0.05; 95% CI 0.01, 0.11) and 60–64 (MD 0.06; 95% CI 0.009, 0.11).

**Conclusions:**

Cohort members who experienced increasing area disadvantage from childhood were at increased risk of poor mental health over the life course. Population-wide interventions aiming at improving social and physical aspects of the early neighbourhood environment could reduce the socio-economic burden of poor mental health.

**Supplementary Information:**

The online version contains supplementary material available at 10.1007/s00127-023-02427-x.

## Introduction

The life course approach highlights the importance of timing, duration and temporal ordering of effects between exposure and outcomes [1, 2]. The timing of an exposure may be particularly important for mental health during social transitions from childhood to adulthood [3, 4] and for the duration of lifestyle and environmental risk factors which tend to accumulate over the life course [5].

However, these mental health determinants do not only cluster temporally but also spatially [6–10] and there are potential reasons why such a spatio-temporal relationship may exist [3]. Living in a disadvantaged area could impact mental health via scarcity of community resources, such as adverse built environment exposures (e.g., noise [11], air pollution [12]) and through stress exposures (e.g., crime [13]) and these impacts could be modified by individual’s socio-economic status [14, 15]. A non-causal explanation could be selection or ‘social drift’, where people with mental health problems may relocate to disadvantaged areas. These processes may interact to form a chain of cumulative risk [16]. Nevertheless, five systematic reviews [6–10] over the last decade investigating area socioeconomic conditions and mental health concluded that evidence is still inconclusive and that longitudinal designs with extensive area histories are warranted [6, 17].

**Fig. 1:**
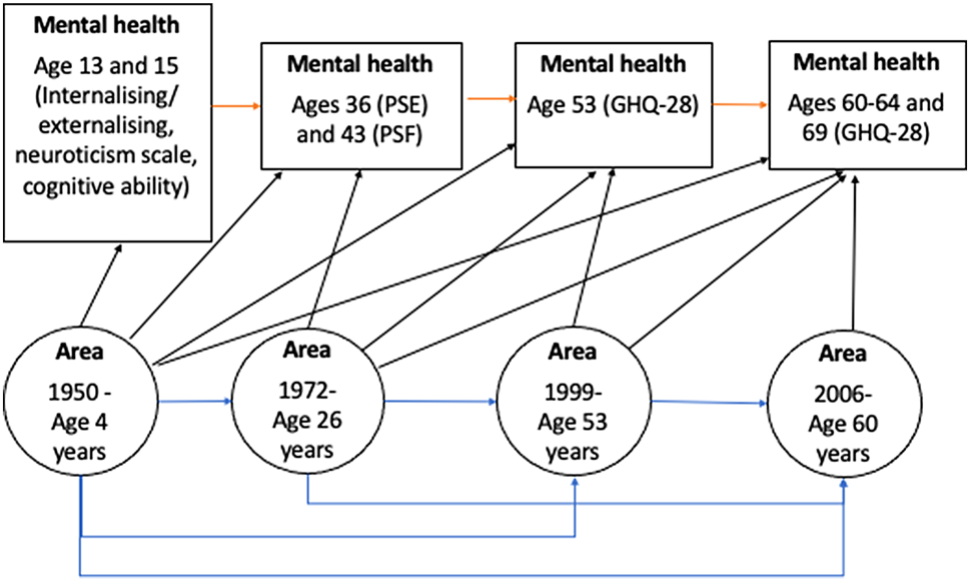
Potential pathways of neighbourhood effect on mental health over the life course. Black pathways refer to potential associations between area disadvantage and mental health. Blue pathways refer to potential continuity in area characteristics. Red pathways refer to continuity in affective symptoms. *PSE* Total score of Present State Examination at age 36. *PSF* Total score of Psychiatric Symptom Frequency scale at age 45 GHQ-28: Total score of 28 item General Health Questionnaire at ages 53, 60–64 and 69

The aim of this study was, therefore, to investigate whether area disadvantage is associated with mental health over a 69-year period using prospectively collected residential addresses from the MRC National Health Survey of Health and Development (NHSD), the British 1946 birth cohort. We investigated the following four hypotheses (Fig. 1): (i) Early area level disadvantage will be associated with poorer adolescent and adult mental health [6]; (ii) Area disadvantage at different stages in adulthood will be associated with poorer adult mental health [18]; (iii) Increasing area level disadvantage over time from childhood will be associated with poorer mental health [3]; (iv) these associations will be exacerbated by participant’s social disadvantage [15].

## Methods

### Study population and design

The MRC NSHD is a socially stratified sample originally consisting of 5362 singleton births during one week in March 1946. Cohort members have been followed up 24 times since birth and a wealth of medical and socioeconomic data has been collected throughout their life. At 69 years, this sample consisted of 2125 men and women (61% of the original cohort) still alive and living in England, Scotland and Wales. The sampling procedure and follow-up have been described in detail elsewhere [19] and the cohort socioeconomic profile was broadly similar to a census reference population at age 60–64 [20]. The most recent ethical approval was granted by the National Research Ethics Service Committee London Queen Square and by the Scotland Research Ethics Committee (REC) (14/LO/1073) and Scotland A REC (14/SS/1009). All study members gave written informed consent and did not receive financial reimbursement. Weights were applied to the analysis to account for the sampling procedure. The sample was distributed geographically in proportion to the national population. This study followed the Strengthening the Reporting of Observational Studies in Epidemiology (STROBE) reporting guideline for cohort studies.

### Life course area disadvantage

At every data collection, the address of the current place of residence of each study member was recorded. Place of residence at four different ages was chosen to represent area in different age periods: in childhood (age 4 years—1950), early adulthood (age 26 years—1972) and middle adulthood (age 53 years—1999 and age 60 years—2006) and to be close to census years (eFigure S1–see online supplement). The overall process of linking residential addresses to area level measures (local authority district in England and Wales; counties in Scotland which is approximately linked to a population of 110 000, similar to county level data in the US) was a two-step one and is described in detail elsewhere [21].

In brief, automated matching, county administrative diagrams and manual methods of assignment was carried out on addresses in order to allocate to each place of residence a grid coordinate [22]. Second, these generated coordinates were used to link area data from the closest census: 1951 data for local government districts for 1950, 1971 data for districts for 1972, 2001 data for districts or unitary authorities for 1999 and 2011 data for census local authorities for 2011 [21, 22]. For 1951 and 1971, Scottish addresses had to be linked to data for counties and the four main cities, since district data were not available [21, 22].

We linked MRC NSHD participants at Local Authority level to two measures of area disadvantage: (i) the proportion of employed persons at ages 4, 26, 53 and 60 in each area with occupations that were semi-skilled or unskilled. This was defined according to the UK Registrar General and selected as the primary marker of area socioeconomic disadvantage, since this was previously shown to be the most consistent and appropriate available census variable in NSHD across all study years [22] (ii) the change score in these proportions of area level disadvantage over time. Specifically, change scores in percentage of area disadvantage were estimated by the difference in percentages of employed persons with occupations that were semi-skilled or unskilled in each area–difference in the percentage between age 4 and 26, difference in percentage between age 4 and 53 and difference in percentage between age 4 and 60. These differences could either be due to the participant moving area or the area itself changing; however, we could not disentangle this due to lack of extensive moving status information.

### Measures of mental health

#### Self-reported mental health scale and teacher ratings at age 13–15

At age 13 years (1959), participants completed the Pintner Aspects of Personality Inventory [23, 24] which includes a 35-item neuroticism scale. At ages 13–15 years, teachers rated behaviour and emotionality using a forerunner of the Rutter A scale [25, 26]. Previous studies using this cohort have created summary measures of these problems by deriving global measures for each from factor analysis, then dividing scores for these into absent, mild and severe based on established centile cuts [27].

#### Interviews at age 36 and 43

At age 36 (1982), a short version of the Present State Examination (PSE), a clinically validated semi-structured interview administrated by trained nurses, was used [28]. A total symptom score was derived. At age 43 (1989), the Psychiatric Symptom Frequency (PSF) scale, an interview-based 23-item scale derived from the PSE, was administered. A total score was calculated [29] were higher scores indicates increased symptoms.

#### Self-reported questionnaire at age 53, 60–64 and 69

Study members completed the 28-item self-administered General Health Questionnaire (GHQ-28) [30] at ages 53 (1999), 60–64 (2006–2010) and 69 (2015). Each individual item was scored using a 4-point Likert scale, and a log-transformed total score of the 28-item GHQ was produced for each different age were higher scores indicate probable mental ill health.

### Confounders

The following variables were treated as potential confounders: childhood cognitive ability [31] (age 15 years—1961), individual socioeconomic position (SEP) at 3 stages of life: age 4 (1950) using father’s occupational social class; age 36 (1982) and age 53 (1999) using participant’s occupational social class; and educational attainment (up to age 26, i.e., up to 1972). Individual SEP at the three stages of life were selected to match the years for which census data were available. Childhood SEP was based on father’s occupation when the cohort member was aged 4 years. SEP for each time point was fitted as categorical indicators of professional, intermediate, skilled (non-manual), skilled (manual), partly skilled and unskilled, based on the UK Registrar General classification. Childhood cognitive ability was represented at age 15 years by tests of verbal and non-verbal intelligence (the AH4 test), reading comprehension, and mathematics. Scores were summed to represent overall cognitive ability. Educational attainment was based on the highest educational qualifications and their training equivalents attained by 26 years and were classified as none, vocational only and ordinary secondary (O levels), advanced secondary (A levels), or degree level or equivalent.

A full timeline of data collected is presented in eFigure S1 in the online supplement.

### Statistical analyses

First Hypothesis: Early area level disadvantage will be associated with poorer adolescent and adult mental health

Prospective associations between area disadvantage at age 4 and mental health at age 13–15, 36, 43, 53, 60–64 and 69 were examined by two-level linear and ordinal multilevel single time point models with individuals (level 1) nested within areas (level 2). Initially, models were fitted separately for each measure of mental health over time and area disadvantage at age 4 (model 1). Second, each model was adjusted for childhood SEP from the same year (Model 2), and then further adjusted for cognitive ability (Model 3).

These models where individuals are nested within area in a particular year assume that measuring area of residence at one point in the life course is sufficient to assess the association between area disadvantage and mental health. However, as exposure to area disadvantage can change over the life course, models including only a single time point could provide biased estimates of the effect of area on mental health [32, 33]. Cross-classified models are used when there is no strict hierarchical structure to higher level units and comprise individuals who are nested within a cross-classification of two or more differing hierarchies–in our case, participants nested within a cross-classification of neighbourhoods. In our model, we have up to four classifications relating to the areas (at age 4, 26, 53 and 60) at each census year (eFigure S2 in the online supplement).

Second Hypothesis: Area disadvantage at different stages in adulthood will be associated with poorer adult mental health

Cross-classified models were fitted separately to test prospective associations between area disadvantage (i) at age 26 and mental health at ages 36, 43, 53, 60–64 and 69 (ii) at age 53 and mental health at ages 60–64 and 69 (iii) at age 60 and mental health at age 69.

Third Hypothesis: Increasing area-level disadvantage over time will be associated with poorer adult mental health

Similar cross-classified models were fitted to test each association separately for change score in area disadvantage (i) between age 4 and 26 and mental health at ages 36, 43 (ii) between age 4 and 53 and mental health at age 53 (iii) between age 4 and 60 with mental health at ages 60–64 and 69.

Initially, area disadvantage and change scores in area disadvantage were modelled separately for each year (Model 1). Then, each model was adjusted for adult SEP at age 36 or age 53 (Model 2); further adjusted for childhood SEP (Model 3); further adjusted for educational attainment up to age 26 and cognitive ability at age 15 (Model 4); further adjusted for area disadvantage at previous age (Model 5); further adjusted for mental health at previous age (Model 6) (eTable A).

Fourth Hypothesis: Associations will be exacerbated by participant’s social disadvantage

We also tested possible effect modification of the association between mental health and area disadvantage by individual SEP with the inclusion of an interaction term in the above-mentioned models.

We repeated our statistical analyses using the STATA routine ice, an implementation in STATA of the multiple imputations using chained equations (MICE) and compared our results with the original analysis under the missing at random (MAR) assumption [34]. Data analyses were performed using STATA 14.1 and MLwin 3.04.

A table which summarises the above-mentioned models is provided in the supplemental material (eTable A).

## Results

Our final sample for the analysis was 4873 at age 4, 4231 at age 13–15, 3293 at age 36, 3187 at age 43, 2902 at age 53, 2190 at age 60–64 and 2125 at age 69. The average area disadvantage (percentage of persons employed in semi-skilled or unskilled occupations) of the areas in which cohort members lived was highest in 1950, at 29.3% (IQR, 24.1–35.1), and declined steadily to 25.1% (IQR, 21.0–29.5) in 1972, 19.7% (IQR, 17.6–21.6) in 1999 and 15.5% (12.7, 18.0) in 2006. Descriptive statistics of the sample are presented in Table 1.

First Hypothesis: Is early area level disadvantage associated with poorer adolescent and adult mental health?

**Table 1:** Descriptive statistics of mental health measures, cognitive ability, area disadvantage, childhood SEP, adult SEP and educational attainment

Number (%) unless otherwise stated	
Emotional (internalising) problems at age 13–15 (*n* = 4232)	
Absent	2,114 (49.9%)
Mild	1,566 (37.0%)
Severe	552 (13.04%)
Conduct (externalising) problems at 13–15 (*n* = 4231)	
Absent	3,162 (74.7%)
Mild	771 (18.2%)
Severe	298 (7.0%)
Cognitive ability at age 15 (*n* = 4008)	Median:0.05; IQR (− 0.61, 0.63)
Total Score of Neuroticism scale at age 13–15 (*n* = 3804)	Median:10; IQR (7, 13)
Total Score of Present State Examination scale at age 36 (*n* = 3498)	Median:1; IQR (0, 3)
Total Score of Psychiatric Symptom Frequency scale at age 43 (*n* = 3147)	Median:8; IQR (3, 15)
Total score of 28-item GHQ at age 53 (*n* = 2190)	Median:15; IQR (11, 21)
Total score of 28-item GHQ at age 60–64 (*n* = 2902)	Median:14; IQR (11, 20)
Total score of 28-item GHQ at age 69 (*n* = 2125)	Median:13; IQR (10, 18)
Area disadvantage at ages 4, 26, 53, 60	
Percentage of employed persons in each area with occupations that were partly skilled or unskilled at year 1950—age 4 (*n* = 4873)	Median:29.3; IQR (24.1, 35.1)
Percentage of employed persons in each area with occupations that were partly skilled or unskilled at year 1972—age 26 (*n* = 3609)	Median:25.1; IQR (21.0, 29.5)
Percentage of employed persons in each area with occupations that were partly skilled or unskilled at year 1999—age 53 (*n* = 3481)	Median:19.7; IQR (17.6, 21.6)
Percentage of employed persons in each area with occupations that were partly skilled or unskilled at year 2006–age 60 (*n* = 2637)	Median:15.5; IQR (12.7, 18.0)
Father's Social Class at age 4 (*n* 5362)	
Professional	262 (5.7%)
Intermediate	748(16.3%)
Skilled (Non-Manual)	824 (18.1%)
Skilled (Manual)	1,421(31.1%)
Partly skilled	943(20.6%)
Unskilled	302(6.6%)
Dead	66(1.4%)
Social Class at age 36 (*n* = 2890)	
Professional	228 (7.8%)
Intermediate	881 (30.4%)
Skilled (Non-Manual)	627 (21.7%)
Skilled (Manual)	623 (21.5%)
Partly skilled	429 (20.6%)
Unskilled	102(3.5%)
Social Class at age 53 (*n* = 2744)	
Professional	202 (7.3%)
Intermediate	1,033 (37.6%)
Skilled (Non-Manual)	618 (22.5%)
Skilled (Manual)	484 (17.6%)
Partly skilled	301 (10.9%)
Unskilled	106 (3.8%)
Educational attainment up to age 26 (*n* = 4375)	
None	1,741(39.7%)
Vocational only and ordinary secondary (O levels)	1,201(27.4%)
Advanced secondary (A levels),	1,026 (23.4%)
Degree level or equivalent	407 (9.3%)

There was evidence of a prospective association between area disadvantage at age 4 and the total score of neuroticism scale at age 13–15 (Table 2). After adjusting for childhood SEP and cognitive ability at age 15 (Model 3), for each one percentage increase in area disadvantage at age 4, there was a 0.02 (95% CI 0.001, 0.04) increase in the score of the neuroticism scale.

**Table 2:** Associations of area disadvantage (percentage of employed persons in each area with occupations that were partly skilled or unskilled) at age 4 with total score of neuroticism scale at age 13, cognitive ability and emotional and conduct problems at age 13–15 with the use of two-level nested models

	Model 1	Model 2	Model 3
	Unadjusted	Childhood SEP at age 4	Childhood SEP at age 4 + Cognitive ability at age 15
	MD (95% CI)	MD (95% CI)	MD (95% CI)
Total score of neuroticism scale at age 13–15 *N* = 3501	0.05 ** (0.02, 0.07)	0.03** (0.01, 0.05)	0.02* (0.001, 0.04)
	OR (95% CI)	OR (95% CI)	OR (95% CI)
Emotional (internalising) problems at age 13–15 *N* = 3788	1.01 (0.99,1.01)	0.99 (0.98,1.01)	0.99 (0.98,1.01)
Conduct (externalising) problems at age 13–15 *N* = 3788	1.01 (0.99,1.01)	0.99 (0.98,1.01)	0.99 (0.98,1.01)

*MD* mean difference, *OR* odds ratio, *CI* confidence interval, *SEP* socioeconomic position

^a^Derived from two-level nested models with persons nested within areas with specification of random variation in area residence at age 4; ± *p < 0.05, **p < 0.001

^a^Mean Difference and 95% Confidence Intervals (CI) represent a difference in mental health scores per one percentage increase in area disadvantage. Odds Ratio and corresponding 95% CI represent an increased risk of mental health problems per one percentage increase in area disadvantage

At age 60–64, there was evidence for a negative association between area disadvantage at age 4 and the total score of GHQ-28 (MD -0.06; 95% CI -0.12, -0.01) after adjusting for childhood SEP at age 4, adult SEP at age 36 and 53, cognitive ability at age 15 and educational attainment (eTable S3-see online supplemental material). This pattern of a negative association, although weaker, between area disadvantage at age 4 and mental health was also observed at ages 36 (eTable S1), 43 (eTable S1) and 53 (eTable S2). No evidence of associations was seen with teacher-rated emotional or conduct problems at ages 13–15 (Table 2) and total score of GHQ-28 at age 69 (eTable S4).

Second Hypothesis: Is area disadvantage at adult stages of the life course associated with poorer adult mental health?

There was no evidence for an association between area disadvantage at age 26 and PSE score at age 36, total PSF score at age 43 (eTable S1–see online supplemental material) and GHQ-28 total score at age 53 (eTable S2). However, a one percentage increase in area disadvantage at age 26 was associated with increased GHQ-28 total score at age 60–64 (MD 0.06; 95% CI 0.01, 0.10) and age 69 (MD 0.05; 95% CI 0.01, 0.12) (eTable S3 and eTable S4).

There was no evidence for an association between area disadvantage at age 53 and the total GHQ-28 score at age 53, 60–64 and 69 (eTable S2, eTable S3 and eTable S4-see online supplement). Nor there was evidence for an association between area disadvantage at age 60 and total GHQ-28 score at age 60–64 and 69 (eTable S3 and S4–see online supplement).

Third Hypothesis: Is increasing area-level disadvantage over time associated with poorer adult mental health?

**Fig. 2:**
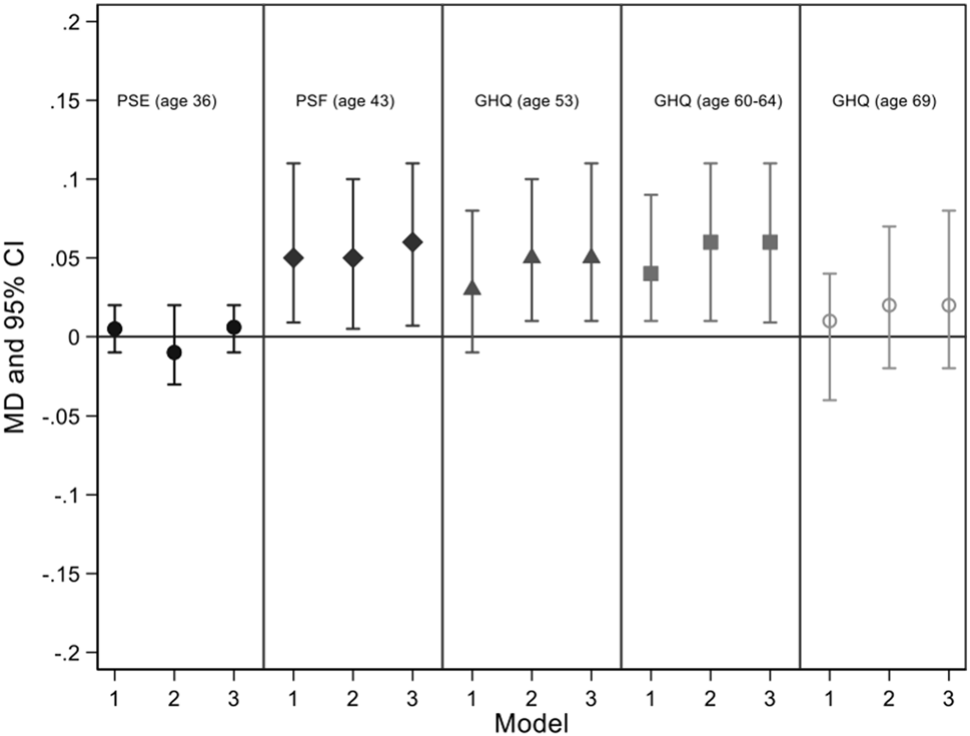
Associations of change score in area disadvantage and adult mental health. Mean Difference (MD) and 95% Credible Intervals (CrI) represent a difference in mental health scores (PSE-age 36; PSF-age 43, GHQ-age 53, age 60–64 and age 69) per one percentage change score increase in area disadvantage between age 4 and 26 (for PSE and PSF), age 4 and 53 (for 28-item GHQ at age 53), age 4 and 60 (for 28-item GHQ at age 60–64 and age 69). In model 1, results were unadjusted; in model 2, results were adjusted for childhood socioeconomic position (SEP) at age 4 and adult SEP (age 4 or age 36 or age 53); model 3 was further adjusted for educational attainment up to age 26 and cognitive ability at age 15, *PSE* Total score of Present State Examination, *PSF* Total score of Psychiatric Symptom Frequency Scale *GHQ* Total score of 28-item General Health Questionnaire

There was evidence of an association between change score in area disadvantage between age 4 and 26 and total score of the PSF at age 43, but not at age 36 (Fig. 2 and Table S1). For example, when adjusting for childhood SEP at age 4, adult SEP at age 36, educational attainment and cognitive ability at age 15 (Fig. 2, model 2), there was a 0.06 (95% CI 0.007,0.11) mean increase in the total score of the PSF per one percentage change score increase in area disadvantage.

In addition, there was evidence of a mean increase (in the total score of the 28-item GHQ at age 53 MD: 0.05; 95% CI 0.01, 0.11) for one percentage change score increase of area disadvantage between age 4 and age 53 (eTable S2). A one percentage change score increase in area disadvantage between age 4 and 60 was also associated with GHQ-28 total score at age 60–64 (MD 0.06; 95% CI 0.009, 0.11) but not with GHQ-28 total score at age 69 (eTable S3 and S4).

Fourth Hypothesis: Are associations between area disadvantage and mental health exacerbated by participant’s social disadvantage?

Effect modification by individual SEP (manual vs non-manual class) of the association between area disadvantage and mental health at ages 13–15 and 69 were observed (eTable S5-see online supplemental material). There were mean differences between individuals from manual and non-manual social classes of 0.017 (0.005, 0.024) and 0.001 (0.0001, 0.003) with mental health outcomes at age 13–15 and age 69, respectively, per one percentage increase in area disadvantage–thus, the association between area disadvantage and mental health outcomes was stronger for those in a manual compared with a non-manual social class.

In addition, effect estimates (OR and MD) and corresponding 95% confidence intervals did not change substantially when we replicate our analyses with the use of the MICE procedure (eTable S6).

## Discussion

This is the first population-based study to utilise geographical data linked to prospectively collected address information in childhood, early adulthood and middle adulthood and subsequently link these exposures to mental health in childhood, early adulthood, middle adulthood and later life during a 69-year period. In relation to our first hypothesis, we found that residence in disadvantaged area in childhood (age 4) was associated with poorer mental health outcomes in early adolescence (age 13), controlling for father’s occupational social class; in contrast, no consistent effect and even a negative effect was detected for early adulthood, middle adulthood or later life. In addition, partly consistent with our second hypothesis, we found poorer mental health in middle adulthood (age 60–64) and later life (age 69) for study members who reside in a disadvantaged area as young adults (age 26). We were partly consistent with our third hypothesis, that cohort members that continued to reside or moved into areas which became more disadvantaged between early life and adulthood had poorer mental health in adulthood. Finally, in regards to our fourth hypothesis, we found that the effects of area disadvantage on mental health were more pronounced in participants from a manual social class, providing evidence of greater vulnerability to area disadvantage in early adolescence (age 13–15) and later life (age 69).

### Strengths and limitations of this study

Strengths of the study include an unusually novel and long follow-up period, and prospective data obtained from a national general population birth cohort based on representative sampling across different socioeconomic areas. We included an extensive range of potential mental health outcomes (repeated collection of mental health outcomes from adolescence and spanning 6 decades) linked with geocoded area data for nearly all study members using linkage with census data. Furthermore, combination of repeated collection of neighbourhood exposure preceding measurement of mental health and vice versa is particularly important to address temporal ambiguity and reverse causality issues observed in cross-sectional studies. People may select into or remain against their desire in certain neighbourhoods due to life history, health or simply in the hope that a different school, neighbourhood, or proximity to specialist health care clinicians might improve their child’s well-being [7, 17, 17, 35].

These strengths should, however, be considered alongside important limitations. A key limitation is that different measures of mental health were used within the NSHD which may specifically impair comparability at ages 13–15 to 69–although, there is no reason to anticipate that this would have changed the pattern of associations observed a the thresholds for case-level symptoms were either clinically validated, or, in the case of the adolescent assessments, consistent with a previous percentile-based cut for the most severe symptoms [36]. In addition, at age 13–15, mental health measures were rated by the teacher which could lead to misclassification and potential bias if participants had emotional problems that were unrecognised by teacher, although there is evidence of consistency between teacher ratings and self-reports of psychiatric disorders [37].

In spite of the population-based sampling, there was selective sample attrition of those less socially advantaged and less healthy and of those with poorer mental health itself [38, 39]. However, comparisons with the census data of the sample successfully contacted at 53 years and weighted to adjust for the initial sampling procedure, show that the sample was representative of the general population of similar age in terms of sex and social class profiles, similar to the 2001 England Census and Integrated Household Survey (IHS) [19, 20]. The greatest overall attrition occurred in the early adult years. In addition, multiple imputation showed very similar associations for the models employed.

The current study utilises administrative boundaries which change over time which may be an imperfect proxy for a person’s true residential area. Furthermore, percentage in skilled vs. unskilled manual occupations, although an adequate proxy in this study [22], does not address all aspects of area disadvantage (such as quality and proximity of amenities and crime) and future studies are needed to identify appropriate conceptualization of place [40] and different area definitions and measurement processes lead to different analytical results [41].

Although we examined a large number of associations and the significance level at 5% is purely nominal and the likelihood of type I error is inflated, the majority of our findings were consistent in terms of the direction of the association between area disadvantage and mental health. In addition, the use of change scores as a measure of area disadvantage should be interpreted with caution as there is a danger of conditioning for variables that are on the causal pathway and further analytical approaches (e.g., causal mediation) should be considered [42].

### Comparison to other studies and discussion of potential mechanism

Our findings provide empirical support to the notion that the child’s environment has a measurable effect on adolescence mental health, in line with previous observational [43–46] and quasi-experimental [47, 48] studies conducted both in the UK, Europe and US.

Our study also indicated a time-lasting association of area disadvantage with mental health that was observed in early adulthood, midlife and later life. Our findings are partly consistent with a Swedish population cohort study of 1.4 million participants where children and adolescents with stability (as number of movers) in their residential environments were less likely to experience psychotic disorders in early adulthood [49]. In addition, higher rates of poor mental health in more deprived, socially fragmented urban environments might be a consequence of social drift [50–53] as many people may move into cheaper and more disadvantaged areas [54, 55]. The vulnerability hypothesis that we tested provides a framework for exploring plausible mechanisms linking area disadvantage with poor mental health. Individuals of lower SEP may be exposed to more stressors such as air [56] and noise [57] pollution, crime [58] and perceived and actual neighbourhood disorder [59, 60] and may have fewer personal resources to cope with these stressors, which may put them at greater risk of poor mental health. We also observed a consistent counterintuitive association between early life area disadvantage and improved mental health outcomes in adulthood (ages 36, 43, 53, 60–64 and 69) (in contrast with findings in adolescence) which was hard to explain and requires replication.

Although overall our effect sizes and 95% confidence intervals might seem small, we need to highlight that associations were observed for a one percentage increase in area disadvantage. If we considered a 10% increase in area disadvantage—which is plausible based on the changes that took place within that time period—our effect sizes and 95% confidence intervals are consistent with similar studies on the topic [6].

## Conclusion

Our results are consistent with a lifelong association of area disadvantage with poor mental health, where a disadvantaged environment could increase the risk of poor mental health; and these risks could be more pronounced in participants from more disadvantaged individual socioeconomic background. Improving neighbourhood environments is a tractable, though complex issue [7] and therefore measures to fund and facilitate area based interventions, such as jobs skill training seminars or psychosocial support for vulnerable individuals [61] may represent a potentially impactful primary health measure for the prevention of poor population mental health. This study also highlights the importance of efforts that are now required to examine the reasons via causal pathway analysis and appropriate geographical levels and provides evidence to direct future interventions and healthcare services in targeting specific vulnerable populations.

## Supplementary Information

Below is the link to the electronic supplementary material.

Supplementary file1 (DOCX 1072 KB)

## Data Availability

The datasets generated during and/or analysed during the current study are not publicly available. Cohort data comply with ESRC data sharing policies, readers can access data via the UK Data Archive (www.data-archive.ac.uk), through a formal request.
