# Supplementary material for: Area disadvantage and mental health over the life course: a 69-year prospective birth cohort study
Source: Soc Psychiatry Psychiatr Epidemiol. 2023 Feb 9;58(5):735–44. doi: 10.1007/s00127-023-02427-x (PMC10097760; doi:10.1007/s00127-023-02427-x)
Supplement: Supplementary file 1 — Supplementary file1 (DOCX 1072 KB) [file 127_2023_2427_MOESM1_ESM.docx]

**Supplementary Online Content**

**Area disadvantage and mental health over the life course: a 69-year prospective birth cohort study**

Ioannis Bakolis ^1,2^, Emily T. Murray ^3^, Rebecca Hardy^4^, Stephani L Hatch^5^, Marcus Richards^4^

**Contents**

Materials are presented in the order they are referred to within the main manuscript

|  |  |
| --- | --- |
| 3-4 | **eTable A.** Description of two level nested and cross-classified models used in a study of area disadvantage across the life course and mental health, United Kingdom, 1946–2015. |
| 5-6 | **eTable S1.** Associations of area disadvantage (percentage of employed persons in each area with occupations that were partly skilled or unskilled) at age 4, 26 and difference (change score) in area disadvantage between age 4 and 26 (difference in percentage of employed persons in each area with occupations that were partly skilled or unskilled) with total score of Present State Examination (PSE) at age 36 and total score of Psychiatric Symptom Frequency (PSF) scale at age 43 with the use of two-level cross classified models ^a^. Mean Difference and 95% Credible Intervals (CrI) represent a difference in scores per one percentage increase in area disadvantage or per one percentage change score increase in area disadvantage. |
| 7 | **eTable S2.** Associations of area disadvantage (percentage of employed persons in each area with occupations that were partly skilled or unskilled) at age 4, 26 and difference (change score) in area disadvantage between age 4 and 53 (difference in percentage of employed persons in each area with occupations that were partly skilled or unskilled) with total score of 28-item General Health Questionnaire (GHQ) at age 53 with the use of two-level nested and cross classified models. Mean Difference and 95% Credible Intervals (CrI) represent a difference in scores per one percentage increase in area disadvantage or per one percentage change score increase in area disadvantage. |
| 8-9 | **eTable S3.** Associations of area disadvantage (percentage of employed persons in each area with occupations that were partly skilled or unskilled) at age 4, 26, 53 and difference in area disadvantage between age 4 and 60 (difference in percentage of employed persons in each area with occupations that were partly skilled or unskilled) with total score of 28-item General Health Questionnaire (GHQ) at age 60-64 with the use of two-level nested and cross classified models. Mean Difference and 95% Credible Intervals (CrI) represent a difference in scores per one percentage increase in area disadvantage or per one percentage change score increase in area disadvantage. |
| 10-11 | **eTable S4.** Associations of area disadvantage (percentage of employed persons in each area with occupations that were partly skilled or unskilled) at age 4, 26, 53 and 60 and difference (change score) in area disadvantage between age 4 and 60 (difference in percentage of employed persons in each area with occupations that were partly skilled or unskilled) with total score of 28-item General Health Questionnaire at age 69 with the use of two-level nested and cross cross classified models. Mean Difference and 95% Credible Intervals (CrI) represent a difference in change scores per one percentage increase in area disadvantage or per one percentage change score increase in area disadvantage.  . |
| 12-13 | **eTable S5.** Effect modification of the association of area disadvantage (% of employed persons in each area with occupations that were partly skilled or unskilled) with mental health andy individual SEP with the use of two-level nested and two-level cross classified models. |
| 14-15 | **eTable S6.** Associations of area disadvantage and change score in area disadvantage and adult mental health with the use of the mice procedure. Mean Difference (MD) and 95% Credible Intervals (CrI) represent a difference in mental health scores (PSE - age 36; PSF - age 43, GHQ - age 53, age 60-64 and age 69) per one percentage change score increase in area disadvantage between age 4 and 26 (for PSE and PSF), age 4 and 53 (for 28-item GHQ at age 53), age 4 and 60 (for 28-item GHQ at age 60-64 and age 69). |
| 16 | e**Figure S1.** Timeline graph of mental health outcomes and area disadvantage in the National Health and Development Survey |
| 17 | **eFigure S2.** Structure of 2-level nested (A) and cross-classified (B) area effects models used in a study of area disadvantage across the life course and mental health, United Kingdom, 1946–2006. |

**eTable A.**

Description of two level nested and cross-classified models used in a study of area disadvantage across the life course and mental health, United Kingdom, 1946–2015.

| **Measure of area disadvantage** | **Measures of Mental Health** | **Multilevel Modelling framework** |
| --- | --- | --- |
| **First Hypothesis: Early area disadvantage will be associated with poorer adolescent and adult mental health** | | |
| Area disadvantage at age 4 | Total Score of Neuroticism scale at age 13, Emotional (internalising) problems at age 13-15, Conduct (externalising) problems at age 13-15; Total score of Present State examination at age 36; Total score of Psychiatric Symptom Frequency scale at age 42; 28-item General Health Questionnaire (GHQ) at age 53; 28-item General Health Questionnaire (GHQ) at age 60-64; 28-item General Health Questionnaire (GHQ) at age 69 | two-level random intercept nested models with persons nested within areas with specification of random variation in area residence at age 4 |
| **Second Hypothesis: Area disadvantage at different stages in adulthood will be associated with poorer adult mental health** | | |
| Area disadvantage at age 26 | Total score of Present State examination at age 36; Total score of Psychiatric Symptom Frequency scale at age 42; 28-item General Health Questionnaire (GHQ) at age 53; 28-item General Health Questionnaire (GHQ) at age 60-64; 28-item General Health Questionnaire (GHQ) at age 69 | a. two-level random intercept nested models with persons nested within areas with specification of random variation in area residence at age 4  b. two-level cross -classified models with persons nested within areas with specification of random variation in area residence at age 4 and age 26 |
| Area disadvantage at age 53 | 28-item General Health Questionnaire (GHQ) at age 60-64; 28-item General Health Questionnaire (GHQ) at age 69 | a. two-level random intercept nested models with persons nested within areas with specification of random variation in area residence at age 53  b. two-level cross -classified models with persons nested within areas with specification of random variation in area residence at age 4, 26, 53 |
| Area disadvantage at age 60-64 | 28-item General Health Questionnaire (GHQ) at age 69 | a. two-level random intercept nested models with persons nested within areas with specification of random variation in area residence at age 60  b. two-level cross -classified models with persons nested within areas with specification of random variation in area residence at age 4, 26, 53 and 60 |
| **Third Hypothesis: Increasing area-level disadvantage over time will be associated with poorer mental health** | | |
| Difference (change score) in area disadvantage between age 4 and 26 | Total score of Present State examination at age 36; Total score of Psychiatric Symptom Frequency scale at age 42 | two-level cross -classified models with persons nested within areas with specification of random variation in area residence at age 4 and age 26 |
| Difference (change score) in area disadvantage between age 4 and 53 | 28-item General Health Questionnaire (GHQ) at age 53 | two-level cross -classified models with persons nested within areas with specification of random variation in area residence at age 4, 26 and 53 |
| Difference (change score) in area disadvantage between age 4 and 60 | 28-item General Health Questionnaire (GHQ) at age 60-64; 28-item General Health Questionnaire (GHQ) at age 69 | two-level cross -classified models with persons nested within areas with specification of random variation in area residence at age 4, 26, 53 and 60 |
| **Fourth Hypotheses: Associations will be exacerbated by participant’s social disadvantage or sex.** | | |
| Interaction term of area disadvantage with Childhood SEP (manual vs non-manual class) or sex at age 4 | Total Score of Neuroticism scale at age 13  Emotional (internalising) problems at age 13-15, Conduct (externalising) problems at age 13-15; | two-level random intercept nested models with persons nested within areas with specification of random variation in area residence at age 4 |
| Interaction term of area disadvantage at age 26 with adult SEP (manual vs non-manual class) or sex at age 36 | Total score of Present State examination at age 36; Total score of Psychiatric Symptom Frequency scale at age 42 | two-level cross -classified models with persons nested within areas with specification of random variation in area residence at age 4 and age 26 |
| Interaction term of area disadvantage with adult SEP (manual vs non-manual class) or sex at age 53 | age 53; 28-item General Health Questionnaire (GHQ) at age 60-64; 28-item General Health Questionnaire (GHQ) at age 69 | two-level cross -classified models with persons nested within areas with specification of random variation in area residence at age 4, 26, 53, 60 |

**eTable S1.**

Associations of area disadvantage (percentage of employed persons in each area with occupations that were partly skilled or unskilled) at age 4 and age 26 and difference (change score) in area disadvantage between age 4 and 26 (difference in percentage of employed persons in each area with occupations that were partly skilled or unskilled) with total score of Present State Examination (PSE) at age 36 and total score of Psychiatric Symptom Frequency (PSF) scale at age 43 with the use of two-level nested ^a^ and cross classified models^b^. Mean Difference and 95% Credible Intervals (CrI) represent a difference in scores per one percentage increase in area disadvantage or per one percentage change score increase in area disadvantage.

|  | **Model 1:**  **Unadjusted** | **Model 2:**  **Adult SEP at age 36** | **Model 3:**  **Childhood SEP at age 4 +Adult SEP at age 36** | **Model 4:**  **Childhood SEP at age 4 + Adult SEP at age 36 + Educational attainment up to age 26 + Cognitive ability at age 15** | **Model 5:**  **Childhood SEP at age 4 + Adult SEP at age 36+ Educational attainment up to age 26 + Cognitive Ability at age 15 + Area disadvantage at age 4** | **Model 6:**  **Childhood SEP at age 4 + Adult SEP at age 36+ Educational Attainment up to age 26 + Cognitive Ability at age 15 + Area disadvantage at age 4 + mental health in previous age** ⱡⱡ |
| --- | --- | --- | --- | --- | --- | --- |
|  | MD  (95% CrI) | MD  (95% CrI) | MD  (95% CrI) | MD  (95% CrI) | MD  (95% CrI) | MD  (95% CrI) |
| **Total score of PSE at age 36** | | | | | | |
| **Area disadvantage at age 4**^a^  **(n=2619)** | -0.01  (-0.03, 0.03) | -0.01  (-0.03, 0.02) | -0.01  (-.04, .002) | -0.02  (-.04, .001) | - | -0.03ⱡ  (-0.05, -0.001) |
| **Area disadvantage at age 26**^a^  **(n=2598)** | -0.02  (-0.04, 0.06) | -0.02  (-0.03, .0.04) | -0.02  (-0.03, .001) | -0.02  (-03, .004) | -0.01  (-05, .05) | -0.01  (-0.05, 0.005) |
| **Area disadvantage at age 26**^b^  **(n=2598)** | -0.02  (-0.04, 0.005) | -0.02  (-0.04, .0.07) | -0.02  (-0.04, .004) | -0.01  (-04, .007) | -0.01  (-05, .01) | -0.02  (-0.05, 0.004) |
| **Difference (change score) in area disadvantage between age 4 and 26** ^b^  **(n=2598)** | 0.005  (-0.01, 0.02) | 0.005  (-0.01, 0.03) | -0.01  (-0.03, 0.02) | 0.006  (-0.01,0.02) | - | -0.01ⱡ  (-0.05, 0.004) |
| **Total Score of PSF scale at age 43** | | | | | | |
| **Area disadvantage at age 4**^a^  **(n=2509)** | -0.06  (-0.1, 0.007) | -0.06  (-0.12, .0.01) | -0.06  (-.11, .008) | -0.07  (-.13, .01) | - | -0.08ⱡ  (-0.14, -0.01) |
| **Area disadvantage at age 26**^a^  **(n=2414)** | 0.03  (-0.05, 0.11) | 0.02  (-0.05, .0.13) | 0.05  (-0.05, .0.13) | 0.01  (-0.03, 0,14) | 0.002  (-0.04, 0.13) | 0.03  (-0.05, 0.005) |
| **Area disadvantage at age 26**^b^  **(n=2414)** | 0.01  (-0.05, 0.11) | 0.03  (-0.04, .0.11) | 0.02  (-0.05, .0.10) | 0.04  (-0.03, 0,11) | 0.03  (-0.05, 0.11) | 0.01  (-0.06, 0.004) |
| **Difference (change score) in area disadvantage between age 4 and 26** ^b^  **(n=2414)** | 0.05*  (0.009, 0.11) | 0.05*  (0.007, 0.10) | 0.05*  (0.005,0.108) | 0.06*  (0.007,0.11) | - | 0.0004ⱡ  (-0.002,0.003) |

Abbreviations: MD: Mean Difference; CrI: Credible Intervals; SEP, socioeconomic position ^a^ Derived from two-level nested models with persons nested within areas with specification of random variation in area residence at age 4 ^b^Derived from two-level cross -classified models with persons nested within areas with specification of random variation in area residence at age 4 and age 26; ⱡIn this case, Model 6 does not include area disadvantage at age 4 as a covariate ; ⱡⱡ mental health in previous age were total score of neuroticism at age 15 when PSE at age 36 was the outcome and PSE at age 36 when PSF at age 43 was the outcome; *p<0.05; **p<0.001

**eTable S2.**

Associations of area disadvantage (percentage of employed persons in each area with occupations that were partly skilled or unskilled) at age 4, 26 and difference (change score) in area disadvantage between age 4 and 53 (difference in percentage of employed persons in each area with occupations that were partly skilled or unskilled) with total score of 28-item General Health Questionnaire (GHQ) at age 53 with the use of two-level cross classified models ^a^. Mean Difference and 95% Credible Intervals (CrI) represent a difference in scores per one percentage increase in area disadvantage or per one percentage change score increase in area disadvantage.

|  | **Model 1:**  **Unadjusted** | **Model 2:**  **Adult SEP at age 53** | **Model 3:**  **Childhood SEP at age 4+Adult SEP (age 4, 36 and 53)** | **Model 4:**  **Childhood SEP at age 4 + Adult SEP (age 4, 36 and 53) + Education attainment up to age 26 + Cognitive ability at age 15** | **Model 5:**  **Childhood SEP at age 4 + Adult SEP (age 4, 36 and 53) + Educational attainment up to age 26 + Cognitive Ability at age 15 + Area disadvantage at previous age (age 4 or 26)** | **Model 6:**  **Childhood SEP at age 4 + Adult SEP (age 4, 36 and 53) + Educational Attainment up to age 26 + Cognitive Ability at age 15 + Area disadvantage at previous age (age 4 or 26) + PSF at age 43** |
| --- | --- | --- | --- | --- | --- | --- |
|  | MD  (95% CrI) | MD  (95% CrI) | MD  (95% CrI) | MD  (95% CrI) | MD  (95% CrI) | MD  (95% CrI) |
| **Area disadvantage at age 4** ^a^  **(n=2181)** | -0.02  (-0.06, 0.02) | -0.02  (-0.08, .0.01) | -0.02  (-0.04, 0.008) | -0.02  (-0.09, 0.009) |  | -0.001ⱡ  (-.003, .03) |
| **Area disadvantage at age 26** ^a^  **(n=2091)** | -0.005  (-0.06, 0.02) | -0.01  (-0.05, 0.04) | -0.01  (-0.09, 0.04) | -0.01  (-0.01, 0.04) | -0.01  (-0.04, 0.01) | -0.01  (-.003, .001) |
| **Area disadvantage at age 26** ^b^  **(n=2091)** | -0.01  (-0.06, 0.05) | -0.01  (-0.07, 0.04) | -0.02  (-0.09, 0.03) | -0.02  (-0.01, 0.03) | -0.02  (-0.05, 0.01) | -0.02  (-.005, .001) |
| **Difference (change score) in area disadvantage between age 4 and 53** ^b^  **(n=2156)** | 0.03  (-0.01, 0.08) | 0.04*  (0.01,0.88) | 0.05*  (0.01,0.10) | 0.05*  (0.01,0.11) | - | 0.002ⱡ  (-.0004, .001) |

Abbreviations: MD: Mean Difference; CrI: confidence interval; SEP: socioeconomic position; PSF: Psychiatric Frequency Scale ^a^ Derived from two-level nested models with persons nested within areas with specification of random variation in area residence at age 4, 26, 53 and 60 ^b^ Derived from two-level cross -classified models with persons nested within areas with specification of random variation in area residence at age 4, 26, 53 and 60

ⱡIn this case, Model 6 does not include area disadvantage at age 4 as a covariate *p<0.05; **p<0.001

**eTable S3.**

Associations of area disadvantage (percentage of employed persons in each area with occupations that were partly skilled or unskilled) at age 4, 26, 53 and difference (change score) in area disadvantage between age 4 and 60 (difference in percentage of employed persons in each area with occupations that were partly skilled or unskilled) with total score of 28-item General Health Questionnaire (GHQ) at age 60-64 with the use of two-level cross classified models ^a^. Mean Difference and 95% Credible Intervals (CrI) represent a difference in scores per one percentage increase in area disadvantage or per one percentage change score increase in area disadvantage.

|  | **Model 1:**  **Unadjusted** | **Model 2:**  **Adult SEP at age 53** | **Model 3:**  **Childhood SEP at age 4 + Adult SEP (4, 36 and 53)** | **Model 4:**  **Childhood SEP at age 4 + Adult SEP (4, 36 and 53) + Education attainment up to age 26 + Cognitive ability at age 15** | **Model 5:**  **Childhood SEP at age 4 + Adult SEP ( age 4, 36 and 53) + Educational attainment up to age 26 + Cognitive ability at age 15 + Area disadvantage at previous age (age 4 or 26 or 53)** | **Model 6:**  **Childhood SEP at age 4 + Adult SEP (age 4, 36 and 53) + Educational attainment up to age 26 + Cognitive ability at age 15 + Area disadvantage at previous age (4 or 26 or 53) + GHQ-28 at age 53** |
| --- | --- | --- | --- | --- | --- | --- |
|  | MD | MD | MD | MD | MD | MD |
|  | (95% CrI) | (95% CrI) | (95% CrI) | (95% CrI) | (95% CrI) | (95% CrI) |
| **Area disadvantage at age 4** ^a^  **(n=1619)** | -0.04  (-0.08, 0.01) | -0.05*  (-0.10, -0.01) | -0.07*  (-0.10, -0.007) | -0.06**  (-0.12, -0.01) |  | -0.004 ⱡ  (-0.008, 0.001) |
| **Area disadvantage at age 26** ^a^  **(n=1615)** | -0.005  (-0.08, 0.03) | 0.01*  (0.01, 0.12) | 0.04*  (0.001, 0.03) | 0.03*  (0.001, 0.10) | 0.01  (-0.01, 0.135 | -0.001  (0.001, 0.001) |
| **Area disadvantage at age 26** ^b^  **(n=1615)** | -0.02  (-0.08, 0.03) | 0.07*  (0.001, 0.13) | 0.07*  (0.01, 0.09) | 0.06*  (0.01, 0.10) | 0.02  (-0.06, 0.05) | -0.002  (-0.008, 0.002) |
| **Area disadvantage at age 53** ^a^  **(n=1612)** | 0.01  (-0.001, 0.001) | -0.01  (-0.01, 0.01) | -0.01  (-0.01, .0.01) | -0.01  (-0.01, 0.01) | -0.01  (-0.001, 0.001) | 0.001  (-0.010, 0.001) |
| **Area disadvantage at age 53** ^b^  **(n=1612)** | 0.02  (-0.10, 0.16) | -0.03  (-0.16, 0.11) | -0.05  (-0.19, .0.09) | -0.04  (-0.19, 0,09) | -0.01  (-0.02, 0.04) | 0.002  (-0.010, 0.001) |
| **Difference (change score) in area disadvantage between age 4 and 60** ^b^  **(n=1611)** | 0.04*  (0.01, 0.09) | 0.05**  (0.01, 0.10) | 0.06*  (0.01, 0 .11) | 0.06*  (0.009, 0.11) | - | 0.003* ⱡ  (0.001, 0.010) |

Abbreviations, Mean Difference; CI, confidence interval; SEP, socioeconomic position

^a^ Derived from two-level nested models with persons nested within areas with specification of random variation in area residence at age 4, 26, 53 and 60 ^b^ Derived from two-level cross -classified models with persons nested within areas with specification of random variation in area residence at age 4, 26, 53 and 60 ⱡIn this case, Model 6 does not include area disadvantage at age 4 as a covariate; *p<0.05 **p<0.001

**eTable S4.**

Associations of area disadvantage (percentage of employed persons in each area with occupations that were partly skilled or unskilled) at age 4, 26, 53 and 60 and difference (change score) in area disadvantage between age 4 and 60 (difference in percentage of employed persons in each area with occupations that were partly skilled or unskilled) with total score of 28-item General Health Questionnaire at age 69 with the use of two-level cross classified models ^a^. Mean Difference and 95% Credible Intervals (CrI) represent a difference in scores per one percentage increase in area disadvantage or per one percentage change score increase in area disadvantage.

|  | **Model 1:**  **Unadjusted** | **Model 2:**  **Adult SEP at age 53** | **Model 3:**  **Childhood SEP at age 4 + Adult SEP (4, 36 and 53)** | **Model 4:**  **Childhood SEP at age 4 + Adult SEP (4, 36 and 53) + Education attainment at age 26 + Cognitive ability at age 15** | **Model 5:**  **Childhood SEP at age 4 + Adult SEP (age 4, 36 and 53) + Educational attainment at age 26 + Cognitive ability at age 15 + Area disadvantage at previous age (age 4 or 26 or 53)** | **Model 6:**  **Childhood SEP at age 4 + Adult SEP (age 4, 36 and 53) + Educational attainment at age 26 + Cognitive ability at age 15 + Area disadvantage at previous age (4 or 26 or 53) + GHQ-28 at age 60-64** |
| --- | --- | --- | --- | --- | --- | --- |
|  | MD | MD | MD | MD | MD | MD |
|  | (95% CrI) | (95% CrI) | (95% CrI) | (95% CrI) | (95% CrI) | (95% CrI) |
| **Area disadvantage at age 4**^a^  **(n=1515)** | 0.02  (-0.04, 0.04) | -0.02  (-0.07, 0.01) | -0.03  (-0.08, .0.01) | -0.03  (-0.08, 0,01) | - | 0.002ⱡ  (-0.001, 0.008) |
| **Area disadvantage at age 26**^a^  **(n=1513)** | 0.001  (-0.10, 0.14) | 0.01*  (0.001, 0.09) | 0.02*  (-0.02, 0.09) | 0.03*  (0.01, 0.11 | 0.03*  (0.01, 0.07) | 0.0001  (-0.001, 0.001) |
| **Area disadvantage at age 26**^b^  **(n=1513)** | 0.06*  (0.08, 0.123) | 0.04*  (0.02, 0.10) | 0.03*  (-0.02, 0.09) | 0.05*  (0.01, 0.12) | 0.04*  (0.01, 0.08) | 0.006  (-0.002, 0.001) |
| **Area disadvantage at age 53**^a^  **(n=1511)** | 0.001  (-0.10, 0.16) | 0.01  (-0.01, 0.01) | 0.02  (-0.10,0.10) | 0.03  (-0.10, 0.09) | 0.02  (-0.18, 0.21) | 0.001  (-0.001, 0.001) |
| **Area disadvantage at age 53**^b^  **(n=1511)** | 0.02  (-0.10, 0.16) | 0.03  (-0.16, 0.11) | 0.04  (-0.10,0.20) | 0.05  (-0.19, 0.09) | 0.03  (-0.20, 0.31) | 0.001  (-0.001, 0.009) |
| **Area disadvantage at age 60**^a^  **n=1474** | 0.02  (-0.05, 0.11) | 0.02  (-0.10, 0.07) | 0.02  (-0.10, 0 .06) | 0.02  (-0.10, 0.08) | -0.01  (-0.15, 0.21) | 0.003  (-0.001, 0.0.11) |
| **Area disadvantage at age 60**^b^  **n=1474** | 0.04  (-0.05, 0.13) | -0.03  (-0.13, .0.06) | -0.03  (-0.13,0 .06) | 0.02  (-0.13, .08) | -0.01  (-0.17, 0.24) | 0.003  (-0.001, 0.0.11) |
| **Difference (change score) in area disadvantage between age 4 and 60**  **(n=1474)** ^b^ | 0.01  (-0.04-0.04) | 0.018  (-0.01, .0.06) | 0.02  (-0.025-0.07) | 0.02  (-0.02, .0.08) | - | 0.004ⱡ  (-0.001, 0.0.11) |

Abbreviations: Mean Difference; CI: confidence interval; SEP: socioeconomic position

^a^ Derived from two-level nested models with persons nested within areas with specification of random variation in area residence at age 4, 26, 53 and 60 ^b^ Derived from two-level cross -classified models with persons nested within areas with specification of random variation in area residence at age 4, 26, 53 and 60ⱡIn this case, Model 6 does not include area disadvantage at age 4 as a covariate; *p<0.05 **p<0.001

**eTable S5.**

Effect modification of the association of early area disadvantage (% of employed persons in each area

with occupations that were partly skilled or unskilled) with mental health by individual SEP with the use

of two-level nested and two-level cross classified models^a^

|  | MD (95% CI) | MD (95% CI) |
| --- | --- | --- |
|  | **Model 1** ^a^**:**  **Childhood SEP at age 4 + Area disadvantage at age 4 + Area disadvantage at age 4 X Childhood SEP at age 4 (manual vs non-manual class)** | **Model 2** ^a^**:**  **Childhood SEP at age 4 + Area disadvantage at age 4 +Area disadvantage at age 4 X Childhood SEP at age 4 (manual vs non-manual class) + Cognitive ability at age 15** |
|  |  |  |
| **Total Score of Neuroticism scale at age 13-15**  **N=3418** | 0.017**±  0.005,0.029 | 0.014  -0.005, 0.024 |
|  | **Model 3** ^b^**:**  **Adult SEP at age 36 + Area disadvantage at age 26 +Area disadvantage at age 26 X Adult SEP at age 36 (manual vs non-manual class)** | **Model 4** ^b^**:**  **Adult SEP at age 36 + Area disadvantage at age 26 +Area disadvantage at age 26 X Adult SEP at age 36 (manual vs non-manual class) +Educational attainment up to age 26 + Cognitive ability at age 15** |
| **Total score of Present state examination scale at age 36**  **N=2606** | -0.01  -0.027, 0.008 | 0.029  -0.020, 0.079 |
| **Total score of Psychiatric frequency scale at age 43**  **n=2421** | 0.007  -0.039, 0.054 | 0.055  -0.087, 0.200 |
|  | **Model 5** ^b^**:**  **Adult SEP at age 53 + Area disadvantage at age 53 +Area disadvantage at age 53 X Adult SEP at age 53 (manual vs non-manual)** | **Model 6** ^b^**:**  **Adult SEP at age 53 + Area disadvantage at age 53 +Area disadvantage at age 53 X Adult SEP at age 53 (manual vs non-manual) +Educational attainment up to age 26 + Cognitive ability at age 15** |
| **Total score of 28-item GHQ score at age 53**  **n=2098** | -0.001  -0.003, 3.40E-06 | -0.002  -0.003, 0.001 |
|  | **Model 7** ^b^**:**  **Adult SEP at age 53 + Area disadvantage at age 60 +Area disadvantage at age 60 X Adult SEP at age 53 (manual vs non-manual)** | **Model 8** ^b^**:**  **Adult SEP at age 53 + Area disadvantage at age 60 +Area disadvantage at age 60 X Adult SEP at age 53 (manual vs non-manual) +Educational attainment up to age 26 + Cognitive ability at age 15** |
| **Total score of 28-item GHQ score at age 63**  **n=1573** | -0.001  -0.003, 0.001 | -0.001  -0.0032, 0.0006 |
| **Total score of 28-item GHQ score at age 69**  **n=1514** | 0.001*  0.0001, 0.003 | 0.001*  0.0001, 0.033 |
|  |  |  |

Abbreviations: MD: Mean Difference; CI, confidence interval; SEP, socioeconomic position

^a^ Derived from two-level cross -classified models with persons nested within areas with specification of random variation in area residence at age 4, 26, 53, and 60.

± The value of the regression coefficient indicates the presence or absence of an interaction of area level SEP and individual’s SEP. For example, a positive value of 0.017 of the regression coefficient indicates that individuals with manual class status have a difference in the mean of 0.017 of total neuroticism score compared to individual with non-manual class status per percentage increase in area disadvantage. *p<0.05 **p<0.001

**eTable S6.**

**(Multiple imputations with chained equations)** Associations of area disadvantage (percentage of employed persons in each area with occupations that were partly skilled or unskilled) and difference (change score) in area disadvantage (difference in percentage of employed persons in each area with occupations that were partly skilled or unskilled) with total score of mental health measures at age 13-15, 36, 43, 53, 60-64, 69 with the use of two-level nested ^a^ and cross classified models^b^. Mean Difference and 95% Credible Intervals (CrI) represent a difference in scores per one percentage increase in area disadvantage or per one percentage change score increase in area disadvantage.

|  | **Total score of Neuroticism Scale**  **at age 13-15**  **(N=4231)** ^c^ | | **Total score of PSE at age 36**  **(N=3293)** ^d^ | | **Total Score of PSF scale at age 43**  **(N=3187)** ^d^ | | **Total score of 28-item General Health Questionnaire (GHQ) at age 53**  **(N=2902)** ^e^ | | **Total score of 28-item General Health Questionnaire (GHQ) at age 60-64**  **(n=2190)** ^e^ | | **Total score of 28-item General Health Questionnaire (GHQ) at age 69**  **(n=2125)** ^e^ | | |
| --- | --- | --- | --- | --- | --- | --- | --- | --- | --- | --- | --- | --- | --- |
|  | | MD | | MD | | MD | | MD | | MD | | MD |  |
|  | | (95% CrI) | | (95% CrI) | | (95% CrI) | | (95% CrI) | | (95% CrI) | | (95% CrI) |  |
| **Area disadvantage at age 4**^a^ | | 0.06 **  (0.03, 0.07) | | -0.01  (-.03, .001) | | -0.02  (-.07, .001) | | -0.01  (-0.05, 0.005) | | -0.02**  (-0.005, -0.01) | | -0.002  (-0.02, 0.01) |  |
| **Area disadvantage at age 26**^a^ | | - | | -0.01  (-03, .002) | | 0.02  (-0.01, 0,07) | | -0.01  (-0.01, 0.02) | | 0.04*  (0.008, 0.09) | | 0.03*  (0.02, 0.009) |  |
| **Area disadvantage at age 26**^b^ | | - | | - | | - | | 0.001  (-0.005, 0.02) | | 0.05*  (0.01, 0.09) | | 0.05*  (0.01, 0.09) |  |
| **Area disadvantage at age 53**^a^ | | - | | - | | - | | - | | -0.01  (-0.01, 0.01) | | 0.03  (-0.10, 0.07) |  |
| **Area disadvantage at age 53**^b^ | | - | | - | | - | | - | | -0.03  (-0.09, 0.07) | | 0.03  (-0.10, 0.06) |  |
| **Area disadvantage at age 60**^a^ | | - | | - | | - | | - | | - | | 0.02  (-0.008, 0.08) |  |
| **Area disadvantage at age 60**^b^ | | - | | - | | - | | - | | - | | 0.02  (-0.13, .06) |  |
| **Difference (change score) in area disadvantage between age 4 and 26**  ^b^ | | - | | 0.0001  (-0.01,0.01) | | 0.07*  (0.03,0.11) | | - | | - | | - |  |
| **Difference (change score) in area disadvantage between age 4 and 53** ^b^ | | - | | - | | - | | 0.05*  (0.01,0.09) | | - | | - |  |
| **Difference (change score) in area disadvantage between age 4 and 60**^b^ | | - | | - | | - | | - | | 0.06*  (0.03, 0.08) | | 0.02*  (0.001, 0.07) |  |

Abbreviations: Mean Difference; CI: confidence interval; SEP: socioeconomic position

^a^ Derived from two-level nested models with persons nested within areas with specification of random variation in area residence at age 4, 26, 53 and 60 ^b^ Derived from two-level cross -classified models with persons nested within areas with specification of random variation in area residence at age 4, 26, 53 and 60ⱡIn this case. ^c^ adjusted for Childhood SEP at age 4 + Cognitive ability at age 15. ^d^ adjusted for Childhood SEP at age 4 + Adult SEP at age 36 + Educational attainment up to age 26 + Cognitive ability at age 15 ^e^ adjusted for Childhood SEP at age 4 + Adult SEP (age 4, 36 and 53) + Education attainment up to age 26 + Cognitive ability at age 15 *p<0.05 **p<0.0

e**Figure S1.** Timeline graph of mental health outcomes and area disadvantage and socio-economic indicators in the National Health and Development Survey


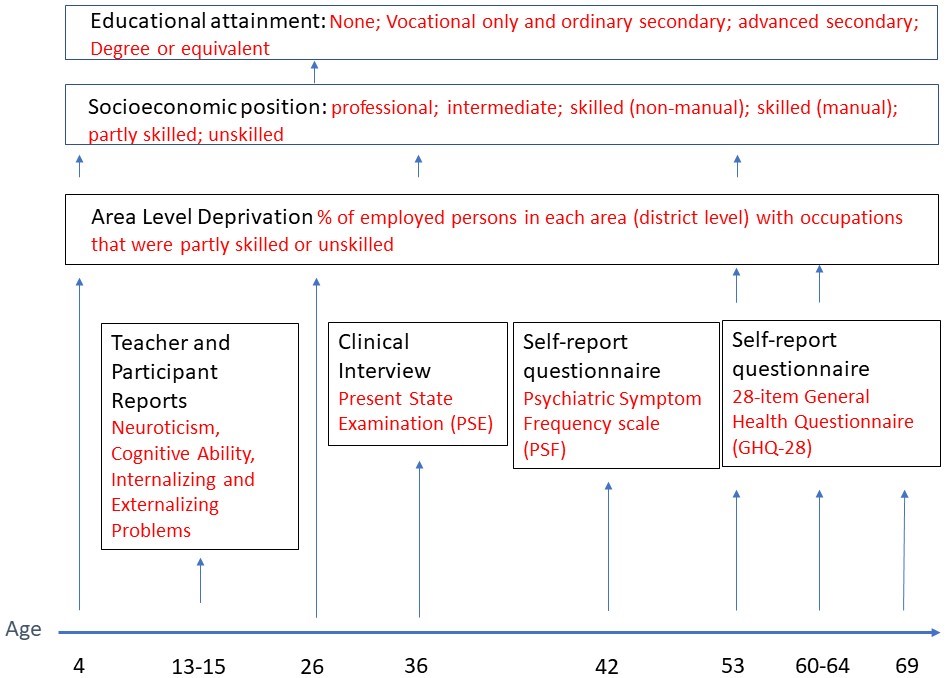


e**Figure S2.** Structure of 2-level nested (A) and cross-classified (B) area effects models used in a study of area disadvantage across the life course and mental health, United Kingdom, 1946–2006.

**
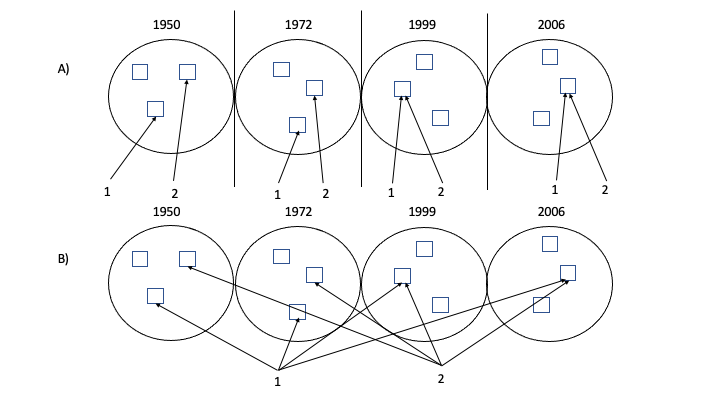
**

Circles are the years in which residential address data were collected; squares are residential areas (local government districts for England and Wales; counties for Scotland). Numbers depict participants 1 and 2. Dashed lines indicate that for 2-level nested models, each year was fitted separately, while for cross-classified models, the absence of dashed lines indicates that all 4 years were fitted in the same model.
